# Factors influencing the intention of doctors to emigrate: a cross-sectional study of Ghanaian doctors

**DOI:** 10.1186/s12913-024-11977-y

**Published:** 2024-11-28

**Authors:** Baleng Mahama Wutor, Francisca Nyarko Sarfo, Louisa Afia Nkrumah, Luki Daniel Bakuoru, Chinenye Nneoma Amanze, Isaac Osei

**Affiliations:** 1 Medical Research Council Unit The Gambia at London School of Hygiene & Tropical Medicine, Fajara, Gambia; 2 Johns Hopkins Bloomberg School of Public Health, Baltimore, USA; 3 Kwame Nkrumah University of Science and Technology, Kumasi, Ghana; 4 University of Energy and Natural Resources, Sunyani, Ghana; 5 Faculty of Infectious and Tropical Diseases, London School of Hygiene & Tropical Medicine, London, UK

**Keywords:** Physician migration, Push and pull factors, Cross-sectional study, Brain drain

## Abstract

**Background:**

The migration of healthcare professionals from developing countries to more developed nations poses a significant challenge to healthcare systems in low- and middle-income countries. This study aimed to determine the proportion of doctors in Ghana who intend to migrate abroad and to identify the sociodemographic and "pull and push" factors that influence their intention.

**Methodology:**

A cross-sectional survey was conducted among doctors in Ghana between March 1, 2024, and March 15, 2024, via an online-based semi-structured questionnaire. Doctors working in Ghana, regardless of nationality, were included. Descriptive statistics and logistic regression analyses were conducted to identify factors associated with the intention to emigrate. Statistical significance was set at a *p*-value of < 0.05.

**Results:**

Almost all the doctors who responded to the questionnaire consented to participate (99.4%, 641/645). More than half (53.8%, n = 345) of the respondents were medical officers. Most respondents intended to migrate to practice abroad (71.8%, n = 460). The United States (59.7%), the United Kingdom (39.1%), and Canada (34.8%) were the most preferred destinations. After adjusting for covariates, young doctors between 20–29 years [(Adjusted Odd Ratios) AOR = 2.69, 95% CI = 1.13—6.39)], male doctors (AOR = 1.53, 95% CI = 1.04—2.25), doctors in lower professional ranks, and doctors in the field of diagnostics (AOR = 5.70, 95% CI = 1.16 – 28.03) had significantly higher odds of intending to migrate. In descending order of magnitude, the respondents strongly agreed that better remuneration (1.22 ± 0.63), better quality of life (1.22 ± 0.67), better working conditions (1.26 ± 0.69), and better postgraduate training (1.41 ± 0.80) were pull factors. The push factors were economic challenges (1.17 ± 0.49), a lack of a conducive working environment (1.56 ± 0.86), slow career progression (1.95 ± 1.07), excessive workload (2.07 ± 0.12), personal circumstances (2.26 ± 1.19), and poor postgraduate training (2.48 ± 1.22).

**Conclusion:**

A substantial proportion of doctors in Ghana are considering emigration, driven by a combination of attractive opportunities abroad and challenging conditions in Ghana. Addressing these issues through improved remuneration, better working environments, and enhanced career development and training opportunities is crucial to retaining healthcare professionals.

**Supplementary Information:**

The online version contains supplementary material available at 10.1186/s12913-024-11977-y.

## Background

Health is a foundational element across various sectors in any nation and is the cornerstone for attaining 12 of the 17 Sustainable Development Goals [1, 2]. It is therefore envisioned that by 2030 all countries will achieve Universal Health Coverage, and every person will have access to quality health care [3]. The attainment of these objectives relies on a well-trained and motivated health workforce. This notwithstanding, the World Health Organization (WHO) projects that Africa will suffer a deficit of 6.1 million health workers by 2030 [4]. The WHO African Region has the lowest number of physicians per population globally [5]. A significant factor contributing to the shortage of healthcare personnel in developing countries and leading to a disproportionate concentration of health workers in developed countries is the migration of doctors from developing countries to developed countries [6–8].

Using data from the American Medical Association, Hagopian and colleagues reported that 23% of physicians practising in the United States received their medical education outside the United States, with the majority (64%) coming from low- and middle-income countries [9]. A blend of macro- (national and global factors), meso- (factors related to professional practice) and micro- (individual-level factors) factors contribute to the intention and actual migration of health workers [10]. These reasons have traditionally been grouped into “pull and push factors”. Pull factors are the conditions in destination countries that make them particularly attractive to people intending to emigrate. Previous studies have identified better remuneration, political stability, opportunities for career progression, the availability of diagnostic tools, and better collaborative working environment as pull factors [10–12]. Push factors are those factors within a country that encourage health workers to emigrate. Factors such as poor remuneration, poor working conditions, high levels of crime, political instability, and slow career progression have been shown by previous studies to contribute to the migration of health workers [12–14].

The pull and push factors are not homogenous. For example, whereas skilled health workers who emigrated from South Africa to the United Kingdom identified insecurity, high crime, and racial tensions as contributing to their decision to migrate, physicians from Nigeria considered poor remuneration to be the most important motivating factor [12, 13, 15]. This highlights the importance of context-specific research on health worker migration. Depending on the prevailing social or economic circumstances in a particular country, the push and pull factors can vary. The phenomenon of Ghanaian doctors emigrating to developed countries is not new [16–18]. However, most of the studies were conducted decades ago and only few studies have specifically investigated the emigration intentions of practicing doctors. Additionally, given the economic challenges Ghana has faced following the COVID-19 pandemic, it is crucial to understand the current emigration intentions of doctors in the country.

This study sought to determine the proportion of doctors living and working in Ghana who intend to emigrate, the sociodemographic factors associated with the intention to emigrate, and the pull and push factors that contribute to their decision. Consistent with terminology used in other studies, the terms “emigration” and “migration” are used interchangeably to refer to the movement of doctors from their countries of origin to practice abroad [19–21].

## Methodology

### Study setting

The study was conducted among doctors in Ghana between March 1, 2024, and March 15, 2024. Ghana is a country in West Africa with a population of 34 million and a Gross Domestic Product per capita of $ 2,238.2 as of 2023 [22]. A quarter of the population lives below $2.15 a day [22]. The healthcare system in Ghana includes public and private health facilities, with disparities in infrastructure and resources between urban and rural areas. Therefore, most doctors in Ghana are concentrated in urban settings. As of 2022, there were only 1.4 medical doctors per 10,000 people in Ghana compared to 8 and 36 medical doctors per 10,000 people in South Africa and the United States respectively [23]. Majority of medical schools in Ghana are publicly funded; therefore, most students pay subsidised tuition fees. After completing medical school, doctors in Ghana receive a provisional license to practice medicine. Following two years of supervised practice, they are fully certified by the Ghana Medical and Dental Council as medical officers. They can then opt to undertake further training to become specialists.

### Study design

We conducted a cross-sectional study among doctors who were living and working in Ghana, regardless of their nationality. A semi-structured online questionnaire (using Microsoft Forms) was developed from a review of the questionnaire used by similar studies (Additional file 1) [11–13, 15, 16]. The questionnaire was piloted among doctors in Ghana. Cronbach's alpha for the 33 items on the Likert scale was 0.83, indicating a satisfactory level of internal consistency in the questionnaire in terms of overall reliability. After validating the questionnaire, it was shared on online messaging platforms (WhatsApp and Telegram) that were exclusive to doctors in Ghana. Reminders were sent out every other day to encourage participation. Before answering the questionnaire, each participant had to read a short introduction that stressed that only doctors currently living and working in Ghana could respond to the questionnaire.

### Sample size determination

According to the Ghana Medical and Dental Council, 11,867 medical doctors have been registered to practise in Ghana [24]. However, this figure includes doctors who have migrated and doctors who may no longer be actively practising medicine. Therefore, the actual number of practising doctors in Ghana is likely to be much lower than this figure. Since this was the only available and verifiable data, we nonetheless used it to calculate the sample size using Cochran’s formula, assuming a confidence interval of 95%, a margin of error of 5%, and an estimated proportion of 50% [25]. A sample size of 373 was derived. However, by the end of the study period, 645 doctors had responded to the survey.

### Data collection and statistical analysis

Data related to sociodemographic factors, the intention of doctors to migrate abroad, and the push and pull factors contributing to their decision-making were collected. The responses were extracted from the online platform and cleaned in Microsoft Excel. STATA (StataCorp, TX, USA) version 18 was used to analyse the data. Descriptive statistics were performed, and the results are presented in tables and bar charts using frequencies and percentages.

We also conducted bivariate logistic regression analyses to examine the associations between sociodemographic factors and the intention of doctors to migrate. Similar to the approach of Onah and colleagues, and to prevent the premature exclusion of potentially important variables, those variables with *p*-values of less than 0.1 were included in a multivariable logistic regression model [12]. The test for statistical significance was set at a *p*-value of < 0.05.

A 5-point Likert scale ranging from strongly agree to strongly disagree was used to assess the level of agreement with the pull and push factors. The average of each factor was calculated. For interpretation, the 5-point Likert scale was converted into classes with equal intervals of 0.80, resulting in the following categories: strongly agree (1.00–1.80), agree (1.81–2.60), neutral (2.61–3.40), disagree (3.41–4.20), and strongly disagree (4.21–5.00).

## Results

### Sociodemographic and professional characteristics of respondents

A total of 645 doctors responded to the survey. Almost all those who responded to the questionnaire consented to participate (99.4%, *n* = 641). The sociodemographic characteristics of the respondents are shown in Table 1. The mean (± SD) age of the respondents was 33 (± 5.8), with most in the age category of 30–39 years (62.1%, *n* = 398). There were more males than females (374 vs 267). Married respondents represented 56.6% (*n* = 363) of the study population. Most respondents had one or more dependents (79.4%, n = 509). Most respondents were brought up in an urban setting (79.4%, *n* = 509) and attended medical school in Ghana (76.0%, *n* = 487). Most doctors (51.0%, *n* = 327) had a monthly income ranging between GHȻ10,000 and GHȻ 15,000.

**Table 1:** Sociodemographic characteristics of respondents

Variable	Frequency (N)	Percentage (%)
**Age category (years)**		
20–29	173	27.0
30–39	398	62.1
40–49	56	8.7
50 and above	14	2.2
Mean age and standard deviation 33 ± 5.8		
**Gender**		
Female	267	41.7
Male	374	58.4
**Marital status**		
Married	363	56.6
Single	278	43.4
**Number of dependents**		
None	132	20.6
1–3	262	40.9
4 or more	247	38.5
**Setting of upbringing**		
Rural	132	20.6
Urban	509	79.4
**Country of medical education**		
Ghana	487	76.0
Outside Ghana	154	24.0
**Monthly income**		
less than GHȻ 5000	20	3.1
GHȻ 5000–7000	53	8.3
GHȻ 7001–9999	177	27.6
GHȻ10000–15000	327	51.0
> GHȻ 15,000	64	10.0

More than half (53.8%, *n* = 345) of the respondents were medical officers and most respondents had been practising medicine for at least three years (84.4%, *n* = 541) (Table 2). Most respondents were general practitioners (38.9%, *n* = 249). The majority of the respondents worked in public health facilities (89.7%, *n* = 575), and most of the health facilities in which they worked were located in urban settings (74.0%, *n* = 474). Regarding the level of health facilities in which the respondents worked, most respondents practised at district hospitals (37.8%, *n* = 242) or teaching hospitals (34.6%, *n* = 222).

**Table 2:** Professional characteristics of respondents

Variable	Frequency (N)	Percentage (%)
**Duration of practice**		
< 2 years	100	15.6
3–5 years	292	45.6
6–10 years	140	21.8
> 10 years	109	17.0
**Professional rank**		
House officer	61	9.5
Medical officer	345	53.8
Resident	115	17.9
Specialist	84	13.1
Senior-specialist/Consultant	36	5.6
**Specialty**		
Medical specialties	361	56.3
Public health/others	16	2.5
Paediatrics	56	8.7
Surgical specialties	193	30.1
Diagnostics	15	2.3
**Work setting**		
Private	66	10.3
Public	575	89.7
**Health facility setting**		
Rural	167	26.1
Urban	474	74.0
**Level of healthcare facility**		
Health centre	12	1.9
District hospital	242	37.8
Private facility	62	9.7
Regional hospital	77	12.0
Teaching hospital	222	34.6
Other	26	4.1

### Migration intention and destination of doctors

**Fig. 1:**
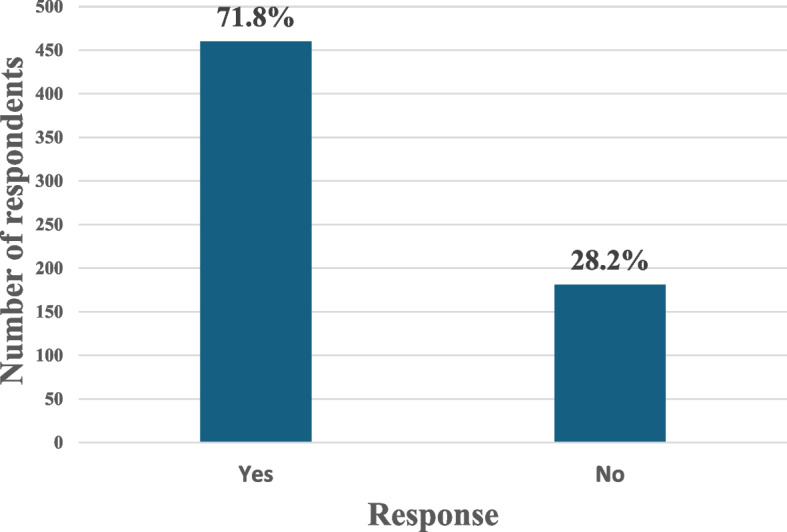
Intention of doctors to migrate abroad

As shown in Fig. 1, most respondents intend to migrate abroad to work as doctors (71.8%, *n* = 460).

**Fig. 2:**
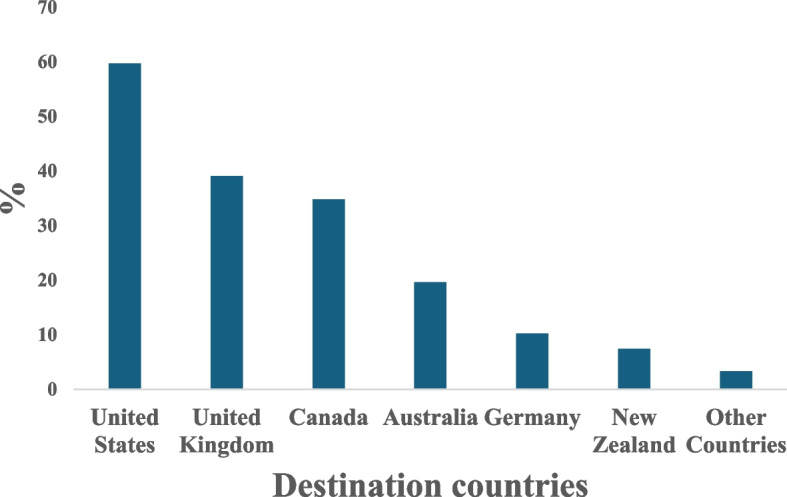
Migration destinations of doctors^a^. ^a^multiple responses were allowed

The migration destinations of doctors who intend to migrate are shown in Fig. 2. The United States (59.7%), the United Kingdom (39.1%) and Canada (34.8%) were the most preferred destinations.

### Sociodemographic factors associated with the intention of doctors in Ghana to migrate

We performed bivariate and multivariable logistic regression analyses of sociodemographic factors associated with the intention to migrate (Table 3). Age category, gender, marital status, country of medical education, professional rank, specialty, and monthly income all had *p*-values < 0.1 in the bivariate analysis and were included in the multivariable logistic regression model. Even though the duration of practice had a *p*-value of less than 0.1, it was excluded from the multivariable logistic regression model due to its collinearity with the age category variable. In the final multivariable logistic regression model, age category, gender, professional rank, and specialty, were significantly associated with intention to emigrate.

**Table 3:** Bivariate and multivariable logistic regression analyses of sociodemographic factors associated with intention to migrate among doctors in Ghana

Variable	Intend to migrate n (%^a^)	Do not intend to migrate n (%^a^)	Bivariate Crude Odds Ratio (95% CI)	*p*-value	Multivariable Adjusted Odds Ratio (95% CI)	*p*-value
**Age Group (years)**						
20–29	142 (82.1)	31 (17.9)	6.48 (3.50—11.99)	** < 0.01**	2.69 (1.13—6.39)	**0.02**
30–39	289 (72.6)	109 (27.4)	3.75 (2.22—6.34)		1.69 (0.874—3.30)	0.12
40 and above	29 (41.4)	41 (58.6)	Ref (1)		Ref (1)	
**Gender**						
Female	181 (67.8)	86 (32.2)	Ref (1)	**0.06**	Ref (1)	
Male	279 (74.6)	95 (25.4)	1.40 (0.99—1.98)		1.53 (1.04—2.25)	**0.03**
**Marital status**						
Married	242 (66.7)	121 (33.3)	Ref (1)	** < 0.01**	Ref (1)	
Single	218 (78.4)	60 (21.6)	1.82 (1.27—2.61)		1.07 (0.68—1.69)	0.76
**Number of dependents**						
None	101 (76.5)	31 (23.5)	1.16 (0.71—1.90)	0.11	NA	
1–3	193 (73.7)	69 (26.3)	Ref (1)			
4 or more	166 (67.2)	81 (32.8)	0.74 (0.50—1.07)			
**Setting of upbringing**						
Rural	101 (76.5)	31 (23.5)	Ref (1)	0.18	NA	
Urban	359 (70.5)	150 (29.5)	0.73 (0.47—1.15)			
**Country where medical degree was obtained**						
Ghana	337 (69.2)	150 (30.8)	Ref (1)		Ref (1)	
Outside Ghana	123 (79.9)	31 (20.1)	1.77 (1.14—2.74)	**0.01**	1.60 (0.98—2.62)	0.06
**Number of years practising medicine**^**b**^						
< 2 years	79 (79.0)	21 (21.0)	5.77 (3.11—10.71)	** < 0.01**	NA	
3–5 years	231 (79.1)	61 (20.9)	5.81 (3.60—9.37)			
6–10 years	107 (76.4)	33 (23.6)	4.98 (2.87—8.62)			
> 10 years	43 (39.5)	66 (60.6)	Ref (1)			
**Professional Cadre**						
House officer	52 (85.3)	9 (14.8)	23.94 (8.05—71.20)	** < 0.01**	7.44 (1.74—31.77)	**0.01**
Medical officer	265 (76.8)	80 (23.2)	13.72 (5.78—32.58)		6.46 (2.04—20.450)	**0.01**
Resident	91 (79.1)	24 (20.9)	15.7 (6.12—40.31)		8.95 (2.89—27.63)	**0.01**
Specialist	45 (53.6)	39 (46.4)	4.78 (1.88—12.15)		3.79 (1.31—10.99)	**0.01**
Senior-specialist/Consultant	7 (19.4)	29 (80.6)	Ref (1)		Ref (1)	
**Speciality area in which you work**				**0.04**		
Medical specialties	269 (74.5)	92 (25.5)	3.76 (1.36—10.41)		1.76 (0.54—5.68)	0.34
Surgical specialty	137 (71.0)	56 (29.0)	3.14 (1.11—8.88)		2.03 (0.62—6.66)	0.24
Diagnostics	12 (80.0)	3 (20.0)	5.14 (1.03—25.71)		5.70 (1.16—28.03)	**0.03**
Pediatrics	35 (62.5)	21 (37.5)	2.14 (0.69—6.63)		1.47 (0.40—5.31)	0.56
Public health/others	7 (43.8)	9 (56.2)	1 (ref)		Ref (1)	
**Type of health facility in which you work**						
Private health facility	49 (74.2)	17 (25.8)	1.15 (0.64—2.06)	0.64	NA	
Public health facility	411 (71.5)	164 (28.5)	Ref (1)			
**Setting of health facility in which you work**						
Rural	124 (74.3)	43 (25.8)	Ref (1)	0.41	NA	
Urban	336 (70.9)	138 (29.1)	0.84 (0.57—1.26)			
**Level of health facility in which you work**						
Health centre	7 (58.3)	5 (41.7)	0.46 (0.14—1.51)	0.40	NA	
District hospital	182 (75.2)	60 (24.8)	Ref (1)			
Private facility	47 (75.8)	15 (24.2)	1.03 (0.53—1.98)			
Regional hospital	55 (71.4)	22 (28.6)	0.82 (0.46—1.47)			
Teaching hospital	153 (68.9)	69 (31.1)	0.73 (0.48—1.10)			
Other	16 (61.5)	10 (38.5)	0.53 (0.22—1.23)			
**What is your monthly income**						
less than GHȻ 5000	15 (75.0)	5 (25.0)	4.68 (1.51—14.53)	** < 0.01**	0.96 (0.26—3.51)	0.95
GHȻ 5000–7000	45 (84.9)	8 (15.1)	8.78 (3.54—21.73)		1.94 (0.64—5.92)	0.24
GHȻ 7001–9999	142 (80.2)	35 (19.8)	6.32 (3.39—11.82)		1.47 (0.61—3.54)	0.39
GHȻ10000–15000	233 (71.3)	94 (28.8)	3.86 (2.21—6.75)		1.28 (0.613—2.69)	0.52
> GHȻ 15,000	25 (39.1)	39 (60.9)	Ref (1)		Ref (1)	

^a^Due to rounding not all categories add up to 100%; CI: Confidence Interval; NA: Not applicable; Ref: Reference

^b^There was collinearity with age category, and it was excluded from the final logistic regression model

Younger doctors between 20–29 years had higher odds of intending to migrate compared with doctors aged 40 years and above (AOR = 2.69, 95% CI = 1.13 – 6.39). Compared with female doctors, male doctors were more likely to have migration intent (AOR = 1.53, 95% CI = 1.04—2.25). Doctors in lower professional ranks had higher odds of intending to migrate than those in higher professional ranks. For instance, the odds of migration intent was 7.4 times higher among house officers (the lowest rank) compared with senior specialists/consultants (AOR = 7.44, 95% CI = 1.74 – 31.77). Compared with doctors working in public health, those working in diagnostics were more likely to have the intent to migrate (AOR = 5.70, 95% CI = (1.16 – 28.03).

### Push and pull factors influencing the intention of doctors in Ghana to migrate

Table 4 shows the average responses to a 5-point Likert scale (1 = strongly agree, 5 = strongly disagree) on the pull and pull factors that contribute to the decision of doctors who expressed the intention to migrate (*n* = 460). On average, the respondents strongly agreed that better remuneration (1.22 ± 0.63), better quality of life (1.22 ± 0.67), better working conditions (1.26 ± 0.69) and better postgraduate training (1.41 ± 0.80) were motivating factors. The following push factors motivated the intention of the respondents to migrate: economic challenges (1.17 ± 0.49), a lack of conducive working environment (1.56 ± 0.86), slow career progression (1.95 ± 1.07), excessive workload (2.07 ± 0.12), personal circumstances (2.26 ± 1.19), and poor postgraduate training (2.48 ± 1.22). However, they disagreed that peer pressure (3.83 ± 1.08) and a high crime rate (3.53 ± 1.13) were motivating factors. There was a neutral response to political instability as a motivating factor (2.84 ± 1.36). Generally, the responses to the pull and push factors were fairly consistent (standard deviation between 0.12 and 1.22).

**Table 4:** Factors that influence the intention of doctors in Ghana to migrate

1. Factors in the destination countries that make them attractive (pull factors) *n* = 460	
	Mean (± SD^b^)
Better remuneration	1.22 (0.63)
Better quality of life	1.22 (0.67)
Better working conditions	1.26 (0.69)
Better postgraduate training	1.41 (0.80)
Peer pressure	3.90 (1.13)
**2. What factors in Ghana motivated your intention to migrate (push factors) *n* = 460**	
	Mean (± SD)
Economic challenges	1.17 (0.49)
Lack of a conducive working environment	1.56 (0.86)
Slow career progression	1.95 (1.07)
Excessive workload	2.07 (0.12)
Personal circumstances	2.26 (1.19)
Poor postgraduate training	2.48 (1.22)
Political instability	2.84 (1.36)
High crime rate	3.53 (1.13)
Peer pressure	3.83 (1.08)
**3. What factors are making you stay and work in Ghana? *n* = 460**	
	Mean (± SD)
Family ties	1.92 (0.98)
Financial challenges involved in emigrating	2.13 (1.03)
Desire to serve Ghana	2.52 (1.04)
Difficulty in obtaining visas	2.75 (1.03)
Difficulty in passing foreign professional examinations	2.80 (0.99)
Racism abroad	2.89 (0.98)
New postgraduate training policy	2.93 (0.92)
Quality of postgraduate training	3.11 (0.79)
Poor remuneration abroad	3.48 (0.78)
Good remuneration	3.52 (0.73)
**4. Will the following factors encourage you to remain and work in Ghana? *n* = 460**	
	Mean (± SD)
Better working conditions	1.49 (1.12)
More stable economy	1.51 (1.14)
Better remuneration	1.54 (1.16)
Better postgraduate training	1.57 (1.08)
Better hospital administration	1.61 (1.14)
Family ties	1.69 (1.01)
Collaborative teamwork among healthcare staff	1.71 (1.08)
Political stability	1.78 (1.09)
Reduced workload	1.98 (1.19)

^b^*SD* Standard deviation, Likert scale interpretation: (Strongly Agree: 1.00–1.80, Agree: 1.81–2.60, Neutral: 2.61–3.40, Disagree: 3.41–4.20, Strongly Disagree: 4.21–5.00. Factors are arranged in descending order of agreement)

### Retention factors

The responses were fairly consistent (standard deviation between 0.73 and 1.19) (Table 3). Regarding the factors motivating respondents to remain and work in Ghana, on average, respondents agreed that family ties (1.92 ± 0.98), financial challenges involved in emigrating (2.13 ± 1.03), and the desire to serve Ghana (2.52 ± 1.04) were motivating factors. However, they were neutral with respect to the new postgraduate training policies (2.93 ± 0.92), the quality of postgraduate training (3.11 ± 0.79), difficulty obtaining visas (2.75 ± 1.03), and racism abroad (2.89 ± 0.98). They disagreed that good remuneration in Ghana (3.52 ± 0.73) and poor remuneration abroad (3.48 ± 0.78) were retention factors.

In descending order, the respondents strongly agreed that the following would encourage them to remain and work in Ghana: better working conditions (1.49 ± 1.12), a more stable economy (1.51 ± 1.14), better remuneration (1.54 ± 1.16), better postgraduate training (1.57 ± 1.08), better administration of hospitals (1.61 ± 1.14), family ties (1.69 ± 1.01), collaborative teamwork among health workers (1.71 ± 1.08), and political stability (1.78 ± 1.09). They also agreed that a reduced workload (1.98 ± 1.19) would encourage them to remain and work in Ghana.

## Discussion

In this study, we investigated the migration intentions of doctors working and living in Ghana, aiming to identify the sociodemographic factors and the push and pull factors influencing their decisions. We found that 71.8% of doctors at various professional ranks in Ghana intended to migrate abroad. Age category, gender, professional rank, and specialty were found to be associated with emigration intention. We also identified fairly consistent push and pull factors that contributed to the decision to migrate. There was strong agreement that better remuneration, better quality of life, better working conditions, and better postgraduate training were pull factors that contributed to their decision to migrate. Economic challenges in Ghana, a lack of a conducive working environment, slow career progression, excessive workload, personal circumstances, and poor postgraduate training were prominent push factors.

The high percentage of doctors intending to migrate in our study is similar to trends seen in Nigeria. For example, a recent study by Akinwumi and colleagues revealed that 74.2% of Nigerian doctors undertaking postgraduate training had the intention to emigrate [19]. Another study conducted among health workers in Nigeria, including doctors, reported that as many as 80.1% of the 513 health workers interviewed intended to emigrate abroad [26]. These similar findings may speak to shared socioeconomic factors in these two countries that contribute to the desire of doctors and health professionals to migrate. However, a previous study by Eliason and colleagues found that only half of medical students in Ghana had migration intentions [27]. Therefore, the socioeconomic conditions of the recent past in Ghana, likely influenced the evolving perspectives of doctors in Ghana. Like other countries, Ghana has encountered numerous economic difficulties in the aftermath of the COVID-19 pandemic, potentially intensifying the desire of many doctors to seek opportunities abroad [28].

In our study, we found fairly consistent strong agreement among doctors that better remuneration, better quality of life, better working conditions, and better postgraduate training were pull factors. On the other hand, a lack of a conducive working environment, slow career progression, excessive workload, personal circumstances, and poor postgraduate training were identified as push factors. Therefore, the positive attributes of destination countries that make them attractive and “pull” doctors almost directly oppose the negative conditions in developing countries that “push” doctors to migrate. The strong agreement for these factors shows their relative importance in influencing the decisions of doctors to migrate or not. In a previous cross-sectional and qualitative study conducted among doctors in Ghana and Nigeria, Hagopian and colleagues also identified similar push and pull factors [29]. Other studies from Nigeria, Liberia, Senegal, and Ethiopia identified comparable push and pull factors [30–33].

Better remuneration emerged as the most significant pull factor, highlighting the disparity in earnings between doctors in Ghana and their counterparts in more developed countries. Doctors are more likely to be retained if they are better remunerated. A previous study by Okeke and colleagues demonstrated how increasing the wages of health workers contributed to reducing the foreign stock of Ghanaian doctors by 10% [34]. Similarly, increasing the wages of South African health workers reduced emigration and even encouraged some emigrants to return [35].

Younger doctors, particularly those in the 20–29 age category, were more likely to express an intention to migrate compared to their counterparts aged 40 years and above. This finding aligns with the results of Onah and colleagues, who observed that Nigerian doctors over the age of 30 were more likely to remain and work in Nigeria compared to their younger counterparts [12]. Akinwumi and colleagues also found that junior resident doctors were more likely to have migration intentions compared to senior resident doctors [19]. Additionally, older doctors, who are likely to have been practicing medicine for a considerable length of time, are more likely to be married and have more dependents. These factors may reduce their motivation to migrate abroad compared to young doctors.

Male doctors had significantly higher odds of intending to migrate compared with their female counterparts. This finding may reflect gender differences in career aspirations, family responsibilities, or opportunities for international mobility. Even though migration within Europe is mainly driven by females, this is less the case in Africa where men still predominate [36]. The difference may also be attributed to gender roles and expectations in Africa, where men might feel more pressure to seek higher-paying opportunities abroad to fulfil financial responsibilities or career ambitions. This notwithstanding, there has been an increase in the number of females, particularly female physicians who are migrating to seek better opportunities abroad [20].

Doctors in the lower professional ranks (house officers, medical officers, and residents) were more likely to have migration intentions compared to senior-specialists/consultants. This could be because early- and mid-career doctors may not feel well compensated or may want to seek opportunities for career progression abroad. It may therefore be apt to provide robust career development pathways within Ghana to retain early-career doctors. It also highlights the importance of having a robust postgraduate medical training program in the country. A study among medical doctors who had just completed their postgraduate training in Ghana showed that doctors would be willing to undertake postgraduate studies and subsequently practise in Ghana if there was an improvement in the training programme [37].

The current strategic plan (2018–2027) of the Ghana College of Physicians and Surgeons (which provides postgraduate training for doctors in Ghana and from the West African subregion) seeks to improve the quality and standards of postgraduate medical training [38]. If this objective is achieved, the college will be able to provide comprehensive and high-quality training opportunities within Ghana, thereby reducing the need for doctors to seek specialised education abroad.

### Strengths and limitations

Our sample size of 641 is one of the highest compared to other studies that have examined the migration intentions of doctors. Our study questionnaire is also comparable to those used in other studies, enabling meaningful comparisons. However, online surveys have inherent sampling and response biases. Since we may not have reached doctors without internet access, there could be some sampling bias. However, this is unlikely given the widespread use of mobile phones in Ghana and generally good internet connectivity across the country [39]. Additionally, even though the questionnaire was shared on platforms that were exclusive to doctors, like any online survey, there is still the possibility of non-doctors being on the platforms and filling them. Regular reminders were also posted on these platforms to discourage participants from submitting multiple responses. Despite these efforts, there is still a chance that some individuals may have filled out the questionnaire more than once. However, during the data cleaning process, no duplicate entries were identified. To further minimize such occurrences, data collection was limited to a 15-day period.

Intentions do not always translate to actual emigration. Therefore, future studies should track the actual emigration of doctors abroad. Further research should also seek to delve deeper into the pull and push factors using qualitative methods to obtain a more nuanced understanding of the intention of doctors to migrate. We are currently analysing the qualitative responses in this study for an independent paper.

### Policy implications and recommendations

Considering the financial resources invested by developing countries in training doctors, their migration abroad is associated with considerable loss in investment for these countries [40]. To retain doctors, it is essential for the Government of Ghana and the Ministry of Health to improve working conditions, offer competitive salaries, and provide professional development opportunities. However, beyond the efforts of individual developing countries, global initiatives are essential to address the macro factors contributing to the migration of doctors [41]. At the international level, it would be helpful to develop comprehensive multilateral policies on the migration of health workers that balance the autonomy of individuals to migrate with the rights of populations and the investment of developing countries in training doctors [30].

## Conclusion

This study highlights the significant intention among doctors in Ghana to emigrate, with 71.8% expressing a desire to migrate abroad. The primary pull factors identified include better remuneration, improved quality of life, better working conditions, and superior postgraduate training opportunities in destination countries. Conversely, the major push factors within Ghana are economic challenges, lack of a conducive working environment, slow career progression, excessive workload, and inadequate postgraduate training in Ghana.

To address the high migration intention of doctors, policymakers in Ghana must focus on improving local working conditions, enhancing remuneration, and providing robust career development pathways and quality postgraduate training programs. Strategies such as increasing wages, improving workplace environments, and fostering a sense of national duty could significantly increase retention rates among doctors. By implementing targeted interventions that address both push and pull factors, Ghana can better retain its medical professionals, ensuring a more stable and effective healthcare system to meet the needs of its population.

## Supplementary Information

Additional file 1. Study questionnaire.

## Data Availability

The datasets used and analysed during this study are available from the corresponding author upon reasonable request.
