# Supplementary material for: Factors influencing the intention of doctors to emigrate: a cross-sectional study of Ghanaian doctors
Source: BMC Health Serv Res. 2024 Nov 28;24:1493. doi: 10.1186/s12913-024-11977-y (PMC11603628; doi:10.1186/s12913-024-11977-y)
Supplement: Supplementary file 1 — Additional file 1. Study questionnaire. [file 12913_2024_11977_MOESM1_ESM.docx]

**Study Questionnaire**

**Study Title: Factors Influencing the Intention of Doctors to Emigrate: A Cross-sectional Study of Ghanaian Doctors**

**Introduction:**

Thank you for offering to fill out this questionnaire, your responses will be treated with strict confidentiality and your participation is entirely voluntary. Your responses will help make clear the factors that may be contributing to the intention of doctors who reside and work in Ghana to emigrate. This questionnaire should be answered by ONLY doctors who reside and work in Ghana.

1. **DEMOGRAPHICS**

***Please tick the appropriate box***

| 1. **Age** (*in years*) | ………………………………….. |
| --- | --- |
| 1. **Gender** | Male  Female  Other, state |
| 1. **Marital Status** | Married  Single. |
| 1. **How many people directly depend on you for their livelihood?** | 1-3  4 or more  None |
| 1. **In which type of setting did you grow up in?** | Rural  Urban |
| 1. **Where did you obtain your MBChB or MBBS?** | Ghana  Other African country  Outside Africa |
| 1. **In what type of medical school did you obtain your MBChB/MBBS?** | Public university in Ghana  Private University in Ghana  University outside Ghana but in Africa  University outside Africa |
| 1. **What is your professional Cadre?** | House Officer  Medical Officer  Resident  Specialist Senior-specialist  Consultant  Other, please specify |
| 1. **How long have you been working as a doctor?** | <2years  3-5 years  6-10 years  >10 years |
| 1. **In which specialty do you currently work?** | Surgery  Obstetrics and gynaecology  Internal medicine  Paediatrics  ENT  Ophthalmology  Anaesthesia  Radiology  Other, please specify |
| 1. **In which setting/sector do you work?** | Public  Private |
| 1. **Do you work in an Urban or rural setting?** | Urban  Rural |
| 1. **What best describes the medical facility in which you work?** | Teaching hospital  Regional hospital  District Hospital  Health centre  CHPS compound  Private facility  Other, specify |
| 1. **What is your monthly income in cedis?** | GHȻ less than 5000  GHȻ 5000-7000  GHȻ 7001-9999  GHȻ10000-15000  >GHȻ15000 |

| 1. **Do you have the intention of migrating out of Ghana to work as a doctor?**   **If “No” Skip to Q 20** | Yes  No |
| --- | --- |
| 1. **Which country do you plan on moving to?** | US  UK  Canada  New Zealand  Australia  Germany  South Africa  Other country, please specify |
| 1. **When did you make the decision to migrate?** | >10 years ago  5-9 years ago  3-4 years ago  1-2 years ago  Less than 1 year ago |
| 1. **What factors in your desired country makes it attractive to you?** | [Likert Scale--Strongly agree to strongly disagree]  Better remuneration for doctors.  Better working conditions  Better post-graduate training  Better quality of life  Peer pressure  Other, please specify |
| 1. **What factors in Ghana motivated your decision/intent to emigrate?** | [Likert Scale--Strongly agree to strongly disagree]  High cost of living  Excessive workload  Economic challenges  Personal circumstances  Peer pressure  High crime rate  Poor post-graduate training/residency  Slow career progression  Political instability  Lack of conducive working environment  Other, please specify |
| 1. **What factors are making you stay and work in Ghana?** | [Likert Scale--Strongly agree to strongly disagree]  Desire to serve Ghana  Family ties  Good remuneration  New post-graduate training policies  Quality of postgraduate training  Difficulty in obtaining visas  Difficulty in passing foreign professional exams  Racism abroad  Poor remuneration abroad  Financial challenges involved in emigrating |
| 1. **Will the following factors encourage you to remain and work in Ghana?** | [Yes or No options for each]  Better remuneration  Better working conditions  Better post-graduate training  A more stable national economy  Collaborative teamwork amongst all healthcare workers |
| 1. **Is there any other thing you believe will encourage the retention of doctors in Ghana?** | Enter text |
